# Effect of the flipped classroom model versus interactive lecture-based learning in undergraduate pediatric education: A crossover study

**DOI:** 10.6026/973206300214056

**Published:** 2025-11-15

**Authors:** Pramila Adhikari

**Affiliations:** 1 Department of Pediatrics, MGM Medical College, Indore, Madhya Pradesh, India

**Keywords:** Medical education, flipped classroom, lecture-based learning, pediatrics, undergraduate teaching

## Abstract

Traditional lecture-based teaching in undergraduate pediatrics often results in passive learning and suboptimal student engagement, necessitating the search for more effective instructional strategies. The present study was an educational intervention study with a crossover design involving 232 third-year medical students. Students were divided into two groups and attended four pediatric topic sessions over five months. In a crossover fashion, each group experienced two topics via FCM (Flipped Classroom Model) and two via LBL (Lecture-Based Learning). The FCM group received pre-reading study materials and 45 45-minute PowerPoint presentation 4 days before class and a case-based scenario was discussed, while the LBL involved faculty-led sessions with integrated questions and discussions. The flipped classroom model proved to be a more effective pedagogical strategy than interactive lectures for improving immediate knowledge acquisition in undergraduate pediatric education.

## Background:

The landscape of medical education is undergoing a fundamental transformation, driven by the global adoption of Competency-Based Medical Education (CBME) frameworks [1]. This paradigm shift emphasizes the development of proficient, self-directed lifelong learners, moving away from traditional didactic lectures towards more active, student-centered learning methodologies [2]. Among these innovative approaches, the flipped classroom model (FCM) has emerged as a particularly promising strategy in health sciences education [3]. The FCM inverts the traditional educational structure: students first acquire foundational knowledge independently through pre-class materials such as video lectures, readings, or online modules. Subsequently, valuable in-class time is repurposed for higher-order cognitive activities, including problem-solving, case-based discussions, and collaborative learning, all under the guidance of a faculty facilitator [4, 5]. This model is theorized to enhance critical thinking, improve knowledge retention, and foster greater student engagement compared to passive learning environments [6]. While the FCM has been increasingly implemented in medical schools, a critical gap persists in the literature. Many studies have demonstrated its superiority over traditional, non-interactive didactic lectures [7, 8]. However, research is scarce directly comparing the FCM to other active learning strategies, such as well-structured interactive lecture-based learning (LBL). Interactive lectures, which incorporate techniques like Socratic questioning, think-pair-share, and polling, are also designed to engage students actively and are often considered a more practical step-up from passive teaching [9]. FCM yields a demonstrable benefit over a well-executed LBL is crucial for curriculum developers. Furthermore, the effectiveness of any pedagogical tool can be context-dependent, varying with the subject matter, learner level, and institutional culture [10]. Therefore, it is of interest to address this gap by directly comparing the effectiveness of the FCM with LBL in the context of undergraduate pediatric teaching.

## Materials and Methods:

## Study design and setting:

A prospective, educational intervention study utilizing a crossover design was conducted over a period of five months from August 2024 to December 2024 in the Department of Paediatrics at MGM Medical College, Indore, a major tertiary care teaching institute in Central India.

## Ethical considerations:

The study protocol was reviewed and approved by the Institutional Ethics Committee of MGM Medical College. Written informed consent was obtained from all participating students after providing them with a detailed explanation of the study's purpose, procedures, voluntary nature of participation, and data confidentiality assurances.

## Study participants:

A total of 232 third-year (Phase 3, Part 1) MBBS students enrolled in the pediatric clinical rotation were recruited for the study.

[1] Inclusion criteria: All third-year MBBS students who provided written informed consent to participate.

[2] Exclusion criteria: Students who did not consent or who failed to complete both the pre-test and post-test for any given session were excluded from the analysis for that specific session's data.

## Procedure:

Upon enrollment, the 232 students were randomly allocated into two groups: Group A (n=116) and Group B (n=116). The study was conducted over four distinct pediatric topics, which were core components of their curriculum (e.g., Neonatal Jaundice, Acute Gastroenteritis, Febrile Seizures, Nutritional Anemia). A crossover design was implemented to ensure each student was exposed to both teaching modalities, thereby minimizing confounding from inherent differences in group academic ability. Also, the perception of each student and faculty was evaluated for the same.

## Flipped classroom model (FCM) arm: 

Four days before the scheduled session, students in this arm received a standardized package of pre-reading learning materials via a shared online drive. This package included a 45-minute narrated PowerPoint presentation, a relevant chapter from a standard pediatric textbook, and one key review article. The 60-minute in-class session was facilitated by a faculty member and focused exclusively on the application of knowledge. Students worked in small groups to solve clinical case scenarios and answer problem-based questions, followed by a large-group discussion to consolidate learning points.

## Interactive Lecture-Based Learning (LBL) arm: 

Students in this arm attended a 60-minute in-class session. The session was delivered by a faculty facilitator using a PowerPoint presentation, but was structured to be highly interactive. Lectures and flip class were taken by different faculties, and a case-based scenario was discussed in the flip class.

## Crossover mechanism: 

For the first topic, Group A was taught using LBL, and Group B was taught using FCM. For the second topic, the methodologies were swapped: Group A received FCM and Group B received LBL. This crossover pattern was repeated for the third and fourth topics.

## Data collection and assessment tool:

Student learning was assessed using a pre-test and a post-test for each of the four topics. The assessment tool for each topic was a set of 10 single-best-answer Multiple-Choice Questions (MCQs), the same for pretest and posttest. These MCQs were developed and validated for content, clarity, and difficulty by three senior faculty members from the Department of Paediatrics. The pre-test was administered at the beginning of each session to assess baseline knowledge, and the identical set of MCQs was administered as a post-test immediately after the session to measure immediate knowledge gain.

## Statistical analysis:

Data were entered into a Microsoft Excel spreadsheet and subsequently analyzed using Microsoft Excel tools. Continuous data (test scores) were presented as mean ± standard deviation (SD). An unpaired t-test was used to compare the mean pre-test and post-test scores between the FCM and LBL groups for each of the four topics and for the aggregate data. A p-value of less than 0.05 was considered statistically significant.

## Results:

Of the 232 students initially enrolled in the study, attendance varied across the four sessions. A total of 120 students (51.7%) attended all four sessions, providing a complete dataset. The remaining students attended a subset of the sessions, and their data were included in the analysis for the specific sessions they completed. Table 1 (see PDF) details the distribution of student attendance. Both the FCM and LBL teaching methods resulted in a significant improvement in knowledge, as evidenced by the increase from pre-test to post-test scores across all four topics. As shown in Table 2 (see PDF), students in both arms of the study demonstrated a positive mean gain in scores, confirming that learning occurred with both pedagogical approaches. The primary analysis compared the effectiveness of FCM versus LBL. The FCM group consistently outperformed the LBL group in both pre-test and post-test scores. As detailed in Table 3 (see PDF), the aggregate mean pre-test score for FCM sessions was significantly higher than for LBL sessions (6.98 vs. 6.39, p=0.001), suggesting students in the FCM arm were better prepared before class. Similarly, the aggregate mean post-test score was also significantly higher for the FCM group (8.50 vs. 8.10, p=0.002), indicating superior knowledge acquisition. On a topic-by-topic basis, post-test scores in the FCM group were statistically significantly higher for Class 1 (p=0.001) and Class 3 (p=0.004).

## Discussion:

This study provides compelling evidence that the flipped classroom model is a more effective pedagogical approach than interactive lecture-based learning for short-term knowledge acquisition in undergraduate pediatric education. The primary finding was the statistically significant superiority of FCM in aggregate post-test scores. This aligns with a growing body of literature supporting the efficacy of FCM in medical education [8, 11]. For example, a study by Patel *et al.* also found that students taught biochemistry via FCM scored significantly better on post-session MCQs than those taught through traditional lectures [12]. Our research extends these findings by demonstrating the FCM's advantage over not just passive lectures, but also a well-established active learning alternative. A particularly noteworthy finding from our study was the significantly higher pre-test scores in the FCM sessions. This suggests that the structured, mandatory nature of the pre-class assignments in the FCM effectively promoted self-directed learning and ensured students arrived in class with a foundational understanding of the topic. This enhanced preparedness is a critical component of the model's success, as it allows in-class time to be dedicated to the higher-order cognitive tasks of application and analysis, as described by Bloom's taxonomy [13]. The LBL group, lacking this structured pre-class component, started from a lower baseline of knowledge. Our results contrast with some studies that have found no significant difference between FCM and other learning methods. For instance, a systematic review by Chen *et al.* concluded that while perceptions of FCM were positive, its effect on academic performance was not consistently superior [14]. Similarly, Prabhu *et al.* found that post-test scores were higher for an interactive lecture group compared to an FCM group in ophthalmology, suggesting that topic complexity and student readiness can influence outcomes [15]. The success of FCM in our study may be attributable to the careful curation of pre-class resources and the strong alignment between pre-class content and in-class case-based application, which is particularly suited to a clinical subject like pediatrics. The implementation of FCM is not without challenges. In our study, significant student attrition was observed, with only about half the cohort attending all four sessions. This is a crucial limitation, as the students who persisted may have been more motivated or academically inclined, potentially biasing the results in favor of a more demanding learning model. This finding underscores the need for institutional support and strategies to encourage student buy-in, as the increased out-of-class workload associated with FCM can be a barrier for some learners [16, 17].

## Limitations:

This study has several limitations. First, its single-center, single-specialty design restricts the generalizability of the findings to other institutions or medical disciplines. Second, the high rate of student dropout may introduce a selection bias. Third, we only measured immediate knowledge acquisition via post-tests; the long-term retention of knowledge and the impact on clinical skills were not assessed.

## Conclusion:

The flipped classroom model demonstrated superior efficacy compared to interactive lecture-based learning in enhancing immediate knowledge acquisition among undergraduate medical students in pediatrics. The model not only resulted in better post-test performance but also significantly improved student preparation before class, fostering a culture of self-directed learning. These findings support the integration of the FCM into medical curricula, particularly for subjects that require strong clinical application skills.
